# The Proteome of Cholesteryl-Ester-Enriched Versus Triacylglycerol-Enriched Lipid Droplets

**DOI:** 10.1371/journal.pone.0105047

**Published:** 2014-08-11

**Authors:** Victor K. Khor, Robert Ahrends, Ye Lin, Wen-Jun Shen, Christopher M. Adams, Ann Nomoto Roseman, Yuan Cortez, Mary N. Teruel, Salman Azhar, Fredric B. Kraemer

**Affiliations:** 1 Division of Endocrinology, Gerontology and Metabolism, Stanford University, Stanford, California, United States of America; 2 Division of Gastroenterology and Hepatology, Department of Medicine, Stanford University, Stanford, California, United States of America; 3 Department of Chemical and Systems Biology, Stanford University, Stanford, California, United States of America; 4 Mass Spectrometry Center, Stanford University, Stanford, California, United States of America; 5 Veterans Affairs Palo Alto Health Care System, Palo Alto, California, United States of America; Simon Fraser University, Canada

## Abstract

Within cells, lipids are stored in the form of lipid droplets (LDs), consisting of a neutral lipid core, surrounded by a phospholipid monolayer and an outer layer of protein. LDs typically accumulate either triacylglycerol (TAG) and diacylglycerol or cholesteryl ester (CE), depending on the type of tissue. Recently, there has been an increased interest in the proteins that surround LDs. LD proteins have been found to be quite diverse, from structural proteins to metabolic enzymes, proteins involved in vesicular transport, and proteins that may play a role in LD formation. Previous proteomics analyses have focused on TAG-enriched LDs, whereas CE-enriched LDs have been largely ignored. Our study has compared the LD proteins from CE-enriched LDs to TAG-enriched LDs in steroidogenic cells. In primary rat granulosa cells loaded with either HDL to produce CE-enriched LDs or fatty acids to produce TAG-enriched LDs, 61 proteins were found to be elevated in CE-enriched LDs and 40 proteins elevated in TAG-enriched LDs with 278 proteins in similar amounts. Protein expression was further validated by selected reaction monitoring (SRM) mass spectrometry (MS). SRM verified expression of 25 of 27 peptides that were previously detected by tandem mass tagging MS. Several proteins were confirmed to be elevated in CE-enriched LDs by SRM including the intermediate filament vimentin. This study is the first to compare the proteins found on CE-enriched LDs with TAG-enriched LDs and constitutes the first step in creating a better understanding of the proteins found on CE-enriched LDs in steroidogenic cells.

## Introduction

Over the past decade, there has been a rise in interest in the interactions of and proteins surrounding intracellular lipid droplets (LDs). A wide array of proteins has been found on the LD surface, from lipid structural proteins to enzymes involved in metabolism, vesicular transport machinery, and several cytoskeletal proteins [1]–[6]. These surrounding proteins have many diverse functions, ranging from LD formation, fusion, binding, and may also serve as markers of cellular signaling [7]. There are two predominant forms of intracellular LDs in mammalian cells, those consisting primarily of triacylglycerol (TAG) and diacylglycerol or those consisting of cholesteryl ester (CE). The type of LD that forms depends on the tissue in which the LD accumulates and the metabolic function of the tissue; adipocytes, liver, and muscle cells accumulate TAG whereas macrophages and steroidogenic cells, such as granulosa and adrenocortical cells, accumulate CE [8].

Recent studies have used genetic and proteomic approaches in identifying and determining the functional role of LD proteins in cellular and LD physiology. Using a genome-wide RNAi screen in *Drosophila* cells, Coat Protein Complex I (COPI) was shown to be required to limit lipid storage and COPI components regulated the composition of perilipins, a family of LD-binding proteins, and promoted the association of adipocyte triglyceride lipase (ATGL) with the LD to mediate lipolysis [9]. In a separate study using an RNAi screen in *Drosophila S2 cells,* 1.5% of all genes were found to function in LD formation and regulation [10]. Enzymes involved in phospholipid biosynthesis were found to affect LD morphology and utilization [10]. Others have approached the role of LD proteins by using proteomic analysis. An earlier study using mass spectrometry (MS) identified LSD2, a *Drosophila* homolog to perilipin, as a regulator of LD transport and homeostasis [11]. Comparative proteomics identified several proteins, including Arfs, Rabs, and lipid synthetic enzymes to be translocated to the LD by GTP-dependent protein recruitment [1]. In cholesterol-loaded macrophages, changes in LD binding proteins were seen between normal chow and western diet fed *Ldlr^−/−^* or *Apoe ^−/−^* mice, suggesting the ability LD proteins to alter cellular function and pathogenesis [2]. Both approaches have highlighted the importance of LD proteins in both cellular and LD physiology.

Among the various proteomics studies that have been published, some proteins are seen in all studies, whereas some proteins are unique to an individual study. In part, this might arise from the fact that different cells and cell lines have been used, with the results being influenced by the fact that certain proteins are expressed in a cell-specific manner. Proteomic studies have used LDs from isolated cells, such as adipocytes, myocytes, or macrophages, or cell lines such as 3T3-L1 and Chinese hamster ovary [3], [4], [12]–[14]. It is expected that certain steroidogenic enzymes would be found on LDs from steroidogenic tissues, such as granulosa, Leydig, and adrenocortical cells and absent in LDs from non-steroid producing cells, such as hepatocytes and skeletal muscle cells. Complications arise when comparing data from *in vitro* loaded cells with *in vivo* generated LDs, since *in vitro* loaded LDs tend to be smaller and multilocular, whereas *in vivo* generated LDs are larger and in the case of adipocytes, unilocular. In addition to variations in tissues and cells, the methods used to identify proteins have varied. Proteins can be separated by SDS-PAGE [3], [14] or 2-D gel electrophoresis [15] and then identified by liquid chromatography/electrospray ionization tandem mass spectrometry (LC/ESI MS/MS). These studies were limited to proteins that were detectable by gel. Alternatively, complex protein mixtures can be identified using gel free proteomics approaches, allowing for global protein identification.

While similarities exist between the two types of LDs, there are also substantial differences between intracellular LDs arising from different tissues. CE-enriched LDs found within steroidogenic tissues tend to be smaller and more numerous than LDs from adipocytes. The functional roles of the LDs are also different; CE-enriched LDs are reservoirs of cholesterol for steroidogenesis, whereas TAG-enriched LDs function in storing energy. Comparisons of protein profiles of CE-enriched and TAG-enriched LDs from different cells would be subject to questions of differences in cellular protein expression. Thus, using the same cell type is important when comparing the protein profiles of CE-enriched and TAG-enriched LDs. Given our interests in the role of LD proteins on steroid production, we chose to use estrogen-primed granulosa cells from naïve rats, cells that have retained their properties of steroidogenesis. Rat granulosa cells were incubated with either high density lipoproteins (HDL) to induce the formation of CE-enriched LDs or fatty acids (FA) to induce the formation of TAG-enriched LDs. Using tandem mass tags (TMT) in combination with LC/ESI MS/MS based proteomics led to 379 LD-associated proteins detected and quantified. Interestingly, while both treatments were to rat granulosa cells, we found 61 proteins to be ≥2-fold higher in CE-enriched LDs and 40 proteins were ≥2-fold higher in TAG-enriched LDs.

## Methods and Procedures

### Ethics Statement

All procedures involving animals were in accordance with institutional and national guidelines and approved by the Institutional Animal Care and Use Committee of the VA Palo Alto Health Care System.

### Experimental animals and granulosa cell isolation

To obtain granulosa cells, 20 naïve 3-week old Sprague Dawley female rats were injected with 1 mg/rat 17β-estradiol subcutaneously for 4 consecutive days. Rats were euthanized on the fifth day and the ovaries were removed. Granulosa cells were isolated by puncturing the ovaries with a 25 gauge needle, pooled, and collected by centrifugation [16]. Granulosa cells were cultured for 72 hrs (DMEM/F12 and 15 mM HEPES and supplemented with 2 µg/ml insulin, 5 µg/ml transferrin, 40 ng/ml hydrocortisone, 150 ng/ml dihydrotestosterone, and 100 U/ml penicillin and 100 µg/ml streptomycin) and then treated with 2.5 mM Bt_2_cAMP for 24 hrs. When cultured with trophic hormones (or second messenger cAMP), these cells become luteinized and, in the presence of lipoprotein, they take in massive amounts of CEs, and respond by producing from 1000–2000 times the progestins (progesterone +20α-hydroxyprogesterone) made by cells grown under basal conditions [17]–[19]. Cells were treated with either 500 µg/ml HDL or 120 µM oleic acid and 120 µM palmitic acid for 48 hrs to induce LD formation, either CE-enriched or TAG-enriched, respectively. Forty-eight hrs incubation was chosen following preliminary experiments that showed optimum LD accumulation along with cell viability at this time.

### Lipid droplet (LD) isolation

LD isolation was adapted from the protocol outlined by Brasaemle et al. [3]. Briefly, cells were scraped from the dish in 25 mM Tris-HCl, 100 mM KCl, 1 mM EDTA, and 5 mM EGTA, pH 7.4 with 100 µM PMSF, 10 µg/ml aprotinin, 10 µg/ml leupeptin, and Halt phosphatase inhibitor cocktail (Pierce, cat # 78420). The cells were frozen and thawed 3 times to lyse cells. The lysate was spun at 1500×g for 10 min to pellet cell debris and nuclei. The lysate was mixed with 37.02% sucrose and overlayed with 9.31%, 4.5%, 0% sucrose in 25 mM Tris-HCl, 1 mM EDTA, 1 mM EGTA with protease and phosphatase inhibitors. The sample was spun at 150,500×g for 60 min. The top fraction was collected and washed 3 times.

### Electron microscopy

Granulosa cells were processed for electron microscopy (EM) by standard techniques as described previously [18]. In brief, cells were fixed for 10 min with 2% glutaraldehyde, scraped from dishes, pelleted by centrifugation and fixed again in glutaraldehyde followed by osmium, uranyl acetate, dehydration in various strengths of alcohol solution, and embedment in plastic. Subsequently, thin sections were stained with uranyl acetate and lead and viewed with a Tecnai G2 Spirit BioTWIN transmission electron microscope from FEI company (Hillsboro, OR) with an AMT DVC camera from Advanced Microscopy Technology Corporation (Woburn, MA). For negative staining of isolated lipid droplets, one drop of the lipid droplet preparation was placed on a formvar coated copper grid. After 1 min the excess material was wicked off and the grid was stained with uranyl acetate (2%, pH 4.5) for 30 sec, air dried, and immediately viewed in the electron microscope [20].

### Measurement of cholesterol and triacylglycerol content

Lipids from granulosa cells were extracted by a modified Folch extraction. Lipids were redissolved in chloroform and loaded onto a TLC plate with cholesterol, cholesteryl ester, and triacylglycerol standards applied on separate lanes, following TLC procedure described previously [21]. The plate was stained with iodine and the spot corresponding to TAG was cut out and eluted. Cholesterol and TAG levels were measured using colorimetric enzymatic assays (Stanbio, Boerne, TX).

### Protein isolation

For proteomics experiments, proteins were precipitated by overnight incubation with 3x volume ice-cold acetone at −20°C, followed by extraction of the proteome with sequential acetone, acetone:ether, and ether treatments. The residual solvent was evaporated under N_2_.

### Protein identification and quantification by tandem mass tags (TMT)

#### Protein digestion

Protein precipitation was performed using acetone on dry ice for 1 hr, followed by ultracentrifugation at 4°C for 10 min. The protein pellet was washed and dried by speedvac. The protein samples were reconstituted in 8 M urea/0.2% protease max (Promega), reduced using DTT at 50°C for 30 min, followed by alkylation using propionamide for 30 min at room temperature. The urea concentration was diluted to less than 1 M by the addition of 50 mM ammonium bicarbonate, pH 8.0. Trypsin was added at a 1∶50 protease to protein ratio and digestion occurred overnight at 37°C.

#### Isobaric tagging

The digestion was quenched by the addition of 10% formic acid water, and peptides were immediately cleaned and concentrated using microspin columns (NEST group). The peptide elute was dried and reconstituted in 100 mM HEPES buffer, pH 8.5. The TMT labels were reconstituted in absolute ethanol and added to the peptide pool following instructions provided with the TMT Mass Tagging Kits and Reagents from Thermo Scientific (Waltham, MA). Protein samples extracted from LDs isolated from granulosa cells loaded with HDL or loaded with fatty acids were labeled with TMT-128 and TMT-129, respectively.

#### Mass spectrometry

Briefly, protein samples were run using a Eksigent 2D nanoLC with buffer A consisting of 0.1% formic acid in water and buffer B consisting of 0.1% formic acid in acetonitrile. A fused silica column, self packed with Ultro120 3 µm C18Q from Peeke Scientific was used with a linear gradient from 5% B to 40% B over 60 min at a flow rate of 750 nl/min. The mass spectrometer was a LTQ-Orbitrap Velos that is set in data dependent acquisition mode to perform MS/MS in HCD mode. The RAW files were searched with a Sorcerer (SageN) processor using the ipi Rat (EMBL) database with a precursor mass tolerance of 50 ppm and later filtered to <25 ppm. At least two peptides for protein assignment are used so as to decrease the false positive discovery rate and add significant values to the quantitative measurements by isobaric tags. Samples were run in duplicate and results were averaged. Using Scaffold Q+ function, protein levels in CE-enriched LDs were expressed as a ratio over TAG-enriched LDs. The data were exported to Excel for further evaluation.

### Sample preparation for mass spectrometry analysis for selected reaction monitoring (SRM)

Proteins were precipitated as above. The pellet was solubilized in 8 M urea for 1 hr with grinding. The sample was diluted to 3 M urea with 100 mM ammonium bicarbonate, pH 8. Reducing agent, tris(2-carboxyethyl)phosphine (TCEP), was added to 10 mM and the sample was shaken for 30 min at 37°C followed by iodoacetamide treatment (final concentration 15 mM) for 30 min at room temperature in the dark. Next, 150 fmol of a heavy peptide per 5 µg of protein was added to allow for quantification of the sample. Trypsin was added (1 µg trypsin/100 µg protein) overnight and the digestion was terminated by acidifying the samples to pH 2–3 with formic acid, desalted on a C18 Sep-Pak cartridge (Waters, Milford, MA, USA), and dried down on a lyophilizer. The peptides were resolubilized in 2% acetonitrile with 0.1% formic acid. The concentration of peptides in each sample was measured at 230 nm using a Nanodrop and adjusted to be 1 µg/µl. Having the same peptide concentration for each sample allowed for more reproducible chromatography, tighter acquisition windows, and thus better signal-to-noise ratio.

### Peptide and SRM transition selection

Proteotypic peptides and transitions (precursor/fragment ions) were selected primarily by screening through the entire sequence of the target protein using unscheduled LC/ESI SRM analysis with the following SRM setup: a scan width of 0.002 m/z was used and a scan time 0.02 s was applied, Q1 and Q3 were set to 0.70 FWHM and the collision gas pressure was of 1.5 Torr. After a set of high quality transitions were found for a peptide (more than 4 transitions that each had a S/N >3), the set was validated using a heavy-labeled synthetic version of each peptide. If the endogenous and the heavy-labeled internal standard peptides showed the same retention time and fragment ion intensity distribution during collision induced fragmentation, the endogenous peptide was selected to be used as a protein probe. For each selected peptide, we chose two or three transitions with the best signal/noise ratio and optimized the collision energy for best sensitivity. The validated and optimized transitions were then used to detect and/or quantify the proteins in lysates using scheduled SRM mode.

### SRM-based quantification

For quantitative analysis, a heavy-labeled synthetic version of each peptide of interest (SpikeTides) was ordered from JPT Peptides (Berlin, Germany). In each peptide the C-terminal K or R residue was substituted with the corresponding heavy version resulting in a mass shift of +8 Da or +10 Da, respectively. The heavy peptides were isotopically-coded, meaning that they co-eluted exactly with the endogenous peptides, and the peak areas could be directly ratioed. The heavy peptides were solubilized in 50 µl of a 20% acetonitrile, 50 mM ammonium bicarbonate solution and combined to a final concentration of 1.6 µM. For each microgram protein digest, 20 fmol of each peptide was used. The peptide transitions in heavy and light versions were measured using scheduled SRM. SRM traces were imported as raw data into Skyline version 2.0 and analyzed. Peak areas for the transitions associated with the heavy and light peptides were quantified by ratioing light and heavy peptide areas. Table S1 displays the peptides utilized and their transitions.

## Results

### LD formation

**Figure 1:**
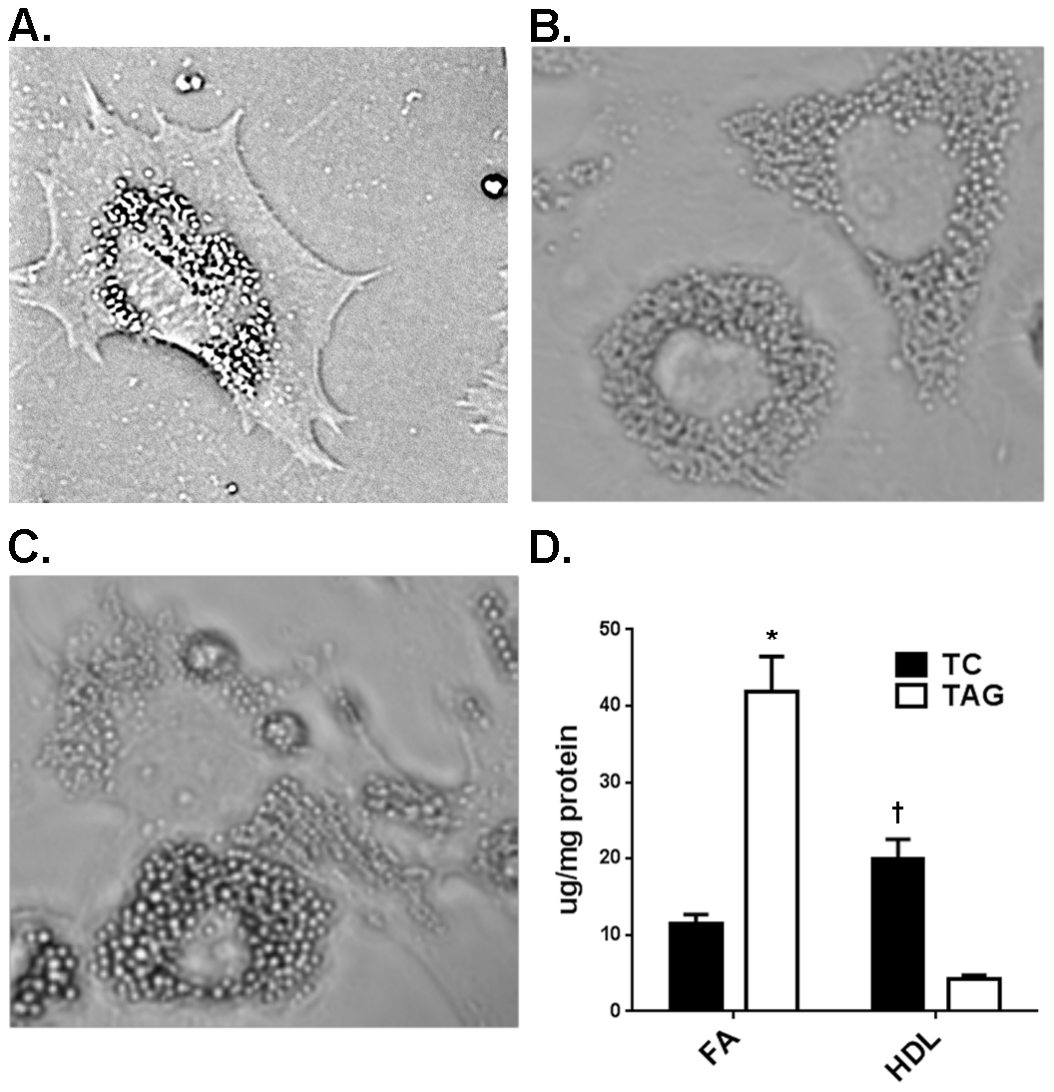
Treatment of cultured granulosa cells with FA or HDL leads to lipid droplet formation. Granulosa cells untreated (**A**) or treated with 240 µM FA (**B**) or 500 µg/ml HDL (**C**) form lipid droplets. **C**. Quantification of total cellular cholesterol (TC) and TAG in granulosa cells following incubation with either FA or HDL. *, p<0.001 versus HDL-loaded; †, p<0.001 versus FA-loaded.

**Figure 2:**
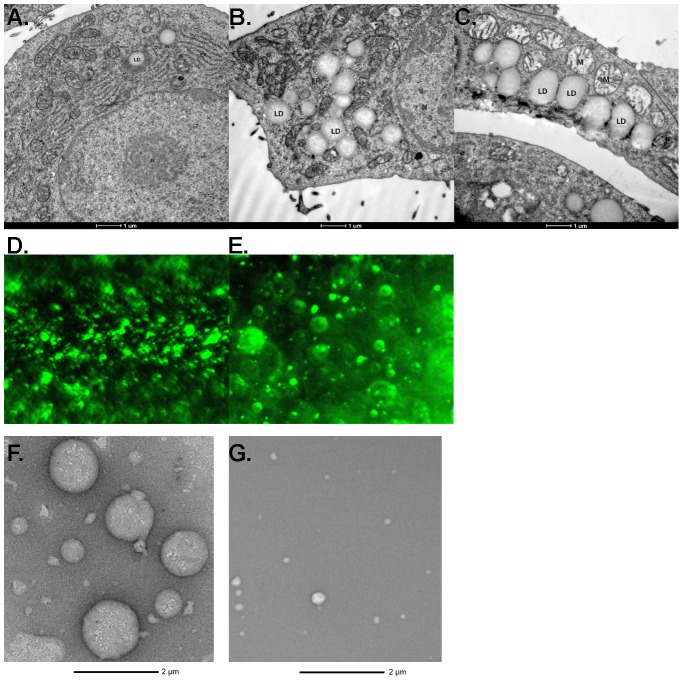
Visualization of lipid droplets. Electron microscopy (magnification 11,500x) of granulosa cells treated with cAMP alone (**A**), with 240 µM FA for 48 h (**B**), or with 500 µg/ml HDL for 48 h (**C**). Fluorescence microscopy of LDs isolated by differential centrifugation from granulosa cells incubated with 240 µM FA for 48 h (**D**), or with 500 µg/ml HDL for 48 h (**E**) followed by BODIPY staining. Electron microscopy (magnification 9,300x) of LDs isolated by differential centrifugation from granulosa cells incubated with 240 µM FA for 48 h (**F**), or with 500 µg/ml HDL for 48 h (**G**). M, mitochondria. LD, lipid droplet, N, nucleus. ER, endoplasmic reticulum.

Granulosa cells from estrogen-primed female naïve rats were untreated (Figure 1A) or treated with either 240 µM FA or 500 µg/ml HDL for 48 hrs to induce the formation of TAG-enriched (**Figure 1B**) or CE-enriched LDs (**Figure 1C**), respectively, as detected by light microscopy. Cellular total cholesterol content was elevated (p<0.001) in cells incubated with HDL to induce formation of CE-enriched LDs, whereas cellular TAG was markedly elevated (p<0.001) in cells incubated with fatty acids to induce TAG-enriched LDs (**Figure 1D**). Using electron microscopy, it is apparent that only rare LDs were detected in cells cultured in the absence of either FA or HDL (**Figure 2A**), whereas abundant LDs are observed in cells incubated either with FA (**Figure 2B**) or with HDL (**Figure 2C**), with the LDs appearing to be in close apposition to mitochondria and ribosomes.

### TMT based proteomics

**Figure 3:**
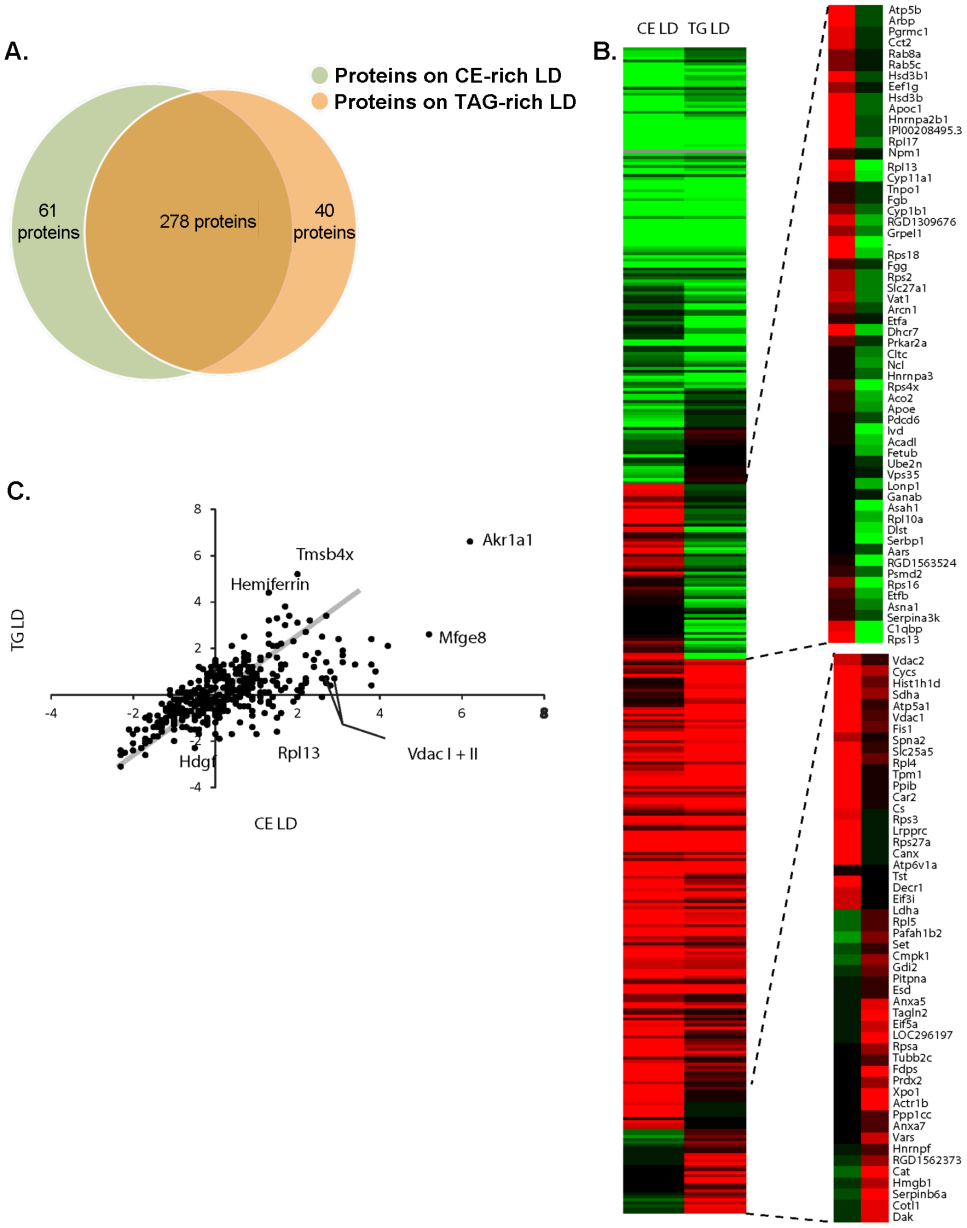
Protein detection in CE-enriched and TG-enriched LDs. **A.** Venn diagram indicating overlap of proteins expressed in CE-enriched and TAG-enriched LDs. **B.** Heat map and representative clustering of CE-enriched LDs compared to TAG-enriched LDs. **C.** Scatter plot of proteome of TAG-enriched LDs and CE-enriched LDs. The scatterplot visualizes the intensity of the tandem mass tag (TMT) reporter channel representing CE against a control channel versus the fold change of the TMT reporter channel representing TAG against the control reporter channel, which are plotted against each other.

LDs were separated from cell membranes, organelles, and cellular debris by centrifugation through a sucrose gradient and then washed 3 times by resuspension in wash buffer. In order to evaluate the purity of the preparations, the isolated LDs were examined by fluorescence microscopy following staining with Bodipy (**Figure 2D** – TAG-enriched, and **Figure** **2E** **–** CE-enriched) and by negative staining electron microscopy (**Figure 2F** – TAG-enriched, and **Figure** **2G** – CE-enriched). While the fluorescence images demonstrate multiple distinct LDs in the preparations isolated from both FA and HDL loaded cells, it is difficult to discern contaminating organelles. The electron micrographic images highlight the spherical structure and variable size of the isolated LDs and demonstrate the apparent absence of any contaminating organelles or membranes in the preparations. Proteins were isolated by acetone precipitation and extracted using acetone, acetone:ether, and ether, resolubilized and digested with trypsin. Peptide samples were then tagged using tandem mass tag labeling and analyzed by LC/ESI MS/MS. There were a total of 379 proteins associated with the LDs that were identified by MS. Similar to previous reports, these proteins consisted of structural proteins, such as Plin2, enzymes involved in various aspects of lipid metabolism, such as fatty acid synthase, vesicular transport machinery, such as several Rab proteins, translational machinery, and several cytoskeletal genes and motor proteins, such as tubulin, vimentin and dynein. Interestingly, 278 proteins were found in relatively equivalent amounts in CE-enriched and TAG-enriched LDs (**Figure 3A**); however based on our TMT quantification results, 61 proteins were observed to be ≥2-fold elevated in CE-enriched LDs and 40 proteins were ≥2-fold enriched in TAG-enriched LDs. **Figure 3B** displays a heat map of the proteins detected in TAG-enriched and CE-enriched LDs. The proteins whose abundance was greater in CE-enriched LDs are listed in **Table 1**, and those proteins whose abundance was greater in TAG-enriched are listed in **Table 2**. The highest enriched protein in CE-enriched LDs was voltage-dependent anion channel 1 (Vdac1). The level was found to be 8-fold higher compared to TAG-enriched LDs. Voltage-dependent anion channel 2 (Vdac2) was also elevated 4.92-fold in CE-enriched LDs compared to TAG-enriched LDs. Other proteins highly elevated in CE-enriched LDs include ADP/ATP translocase (Slc25a5) by 7.46-fold, non-muscle caldesmon (Cald1) by 7.46-fold, myristolyated alanine-rich C-kinase substrate (Marcks) by 6.96-fold, scavenger receptor class B member 1 (Scarb1) by 6.28-fold, 40S ribosomal protein S13 (Rps13) by 6.28-fold, 3 β-hydroxysteroid dehydrogenase/Delta-5, 4- isomerase type 1 (Hsd3b1) by 6.06-fold, lactadherin (Mfge8) by 5.86-fold, ribosomal protein S27a (Rps27a) by 5.66-fold, hepatoma-derived growth factor (Hdgf) by 5.66-fold, and complement component 1Q subcomponent-binding protein (C1qbp) by 5.66-fold. The roles of each of these proteins on the LD are not clear. Several structural proteins were found to be elevated in CE-enriched LDs, vimentin (Vim) by 4.92-fold, myosin-1c (Myo1c) by 4.29-fold, and mysoin-9 (Myh9) by 3.25-fold, suggesting differences in the structural or transporting needs of the LDs. Conversely, proteins elevated in TAG-enriched LDs include thymosin beta-4 (Tmsb4x) by 11.71-fold, hemiferrin by 10.19-fold, catalase (Cat) by 4.92-fold, transgelin (Tagln) by 3.73-fold, 3-alpha-hydroxysteroid dehydrogenase (Akr1c14) by 3.25-fold, zyxin (Zyx) by 3.03-fold, glutathione S-transferase P (Gstp1) by 3.03-fold, and LIM and SH3 domain protein 1 (Lasp1) by 3.03-fold. Catalase was found to be associated, but not in contact, with LDs in 3T3-L1 cells by immunogold staining [22]. Mfge8, hemiferrin, and Tmsb4x were identified to be furthest from the mean by scatter plot (**Figure 3C**).

**Table 1:** List of proteins found in greater abundance in CE-enriched LDs versus TAG-enriched LDs by tandem mass tags based proteomics.

Gene symbol	GI number	Description	CE:TAG
Vdac1	13786200	Voltage-dependent anion channel protein 1	8.00
Slc25a5	32189350	Solute carrier family 25, member 5 (ADP/ATP translocase)	7.46
Cald1	6978589	Caldesmon 1	7.46
Marcks	293356892	Myristoylated alanine-rich C-kinase substrate	6.96
Scarb1	13928730	Scavenger receptor class B member 1	6.28
Rps13	39930505	Ribosomal protein S13	6.28
Hsd3b1	59797058	Hydroxy-delta-steroid dehydrogenase 3β isomerase type 1	6.06
Mfge8	6981200	Milk globule-EGF factor protein 8 protein (lactadherin)	5.86
Rps27a	13592077	Ribosomal protein S27a	5.66
Hdgf	16758528	Hepatoma-derived growth factor	5.66
C1qbp	48675371	Complement component 1 Q subcomponent-binding protein	5.66
Rps19*	82654220	Ribosomal protein S19	5.46
Dhcr7	11693158	7-dehydrocholesterol reductase	5.46
Marcksl1	13540687	MARCKS-like 1	5.28
Rps6	8394224	Ribosomal protein S6	4.92
Vim	14389299	Vimentin	4.92
Hist2h2bb	157820953	Histone cluster 2, H2bb	4.44
Vdac2	13786202	Voltage-dependent anion channel protein 2	4.44
Myo1c	124107592	Myosin-Ic	4.29
Rps18	47087103	Ribosomal protein S18	3.73
Hist1h1d	18959218	Histone cluster 1, H1d	3.73
Hspd1	392343492	Heat shock protein 1	3.73
Atp5a1	40538742	ATP synthase, mitochondrial F1 complex, alpha subunit 1	3.73
Rpl17	42627879	Ribosomal protein L17	3.48
Atp5o	20302061	ATP synthase, mitochondrial F1 complex, O subunit	3.36
Hist2h4	183986773	Histone cluster 2, H4	3.36
Canx	25282419	Calnexin	3.25
Myh9	6981236	Myosin, heavy polypeptide 9, non-muscle	3.25
Ogdh	62945278	Oxoglutarate dehydrogenase	3.25
Fus	58865844	Fused in sarcoma	3.25
Rps4x	56090273	Ribosomal protein S4, X-linked	3.25
Hsd3b2	110835753	Hydroxy-delta-steroid dehydrogenase 3β isomerase type 2	3.25
Rps16	310703682	Ribosomal protein S16	3.03
Apoc1	158517799	Apolipoprotein C-I	3.03
Ddost	58865778	Dolichyl-diphosphooligosaccharide-protein glycosyltransferase	3.03
Atp5b	54792127	ATP synthase, mitochondrial F1 complex, β subunit	2.93
Cyp11a1	8393224	Cytochrome P450, family 11, subfamily a, polypeptide 1	2.83
Mrlc2	203097095	Myosin, light chain 12B, regulatory	2.83
Vapa	118142811	Vesicle-associated membrane protein, associated protein A	2.64
Sdha	18426858	Succinate dehydrogenase subunit A, flavoprotein	2.55
Vat1	76096306	Vesicle amine transport protein 1 homolog	2.55
Phb	13937353	Prohibitin	2.55
Ahnak	300794574	AHNAK nucleoprotein	2.46
Basp1	11560135	Brain abundant, membrane attached signal protein 1	2.46
Rps2	62655115	Ribosomal protein S2	2.46
Grpel1	13324704	GrpE-like 1, mitochondrial	2.38
Spna2	47477769	Spectrin alpha, non-erythrocytic 1	2.38
Car2	9506445	Carbonic anhydrase 2	2.30
Reep5	270288782	Receptor accessory protein 5	2.30
Myh10	149052999	Myosin, heavy polypeptide 10, non-muscle	2.22
Dbn1	13591936	Drebrin 1	2.14
Tapbpl	16975494	TAP-binding protein-like	2.14
Phb2	61556754	Prohibitin 2	2.07
Hadha	148747393	Trifunctional protein, alpha subunit	2.07
Pgrmc1	11120720	Progesterone receptor membrane component 1	2.07
Arbp	11693176	Ribosomal protein, large, P0	2.00
Tpm1	78000194	Tropomyosin 1, alpha	2.00
Rpl6*	162287391	Ribosomal protein L6	2.00
Lrpprc	56605990	Leucine-rich PPR motif-containing	2.00

**Table 2:** List of proteins found in greater abundance in TAG-enriched LDs versus CE-enriched LDs by TMT.

Gene symbol	GI number	Description	CE:TAG
Ldha	8393706	L-lactate dehydrogenase A chain	0.50
Tpi1	117935064	Triosephosphate isomerase	0.50
Gstt2	6980992	Glutathione S-transferase theta-2	0.50
Fabp5	22024394	Fatty acid-binding protein, epidermal	0.50
Pkm2	16757994	Isoform M2 of Pyruvate kinase isozymes M1/M2	0.50
Hdhd2	148540175	Haloacid dehalogenase-like hydrolase domain protein 2 precursor	0.50
Trap1	84781723	TNF receptor-associated protein 1	0.50
Pnp	157822819	Purine nucleoside phosphorylase	0.50
Gmfb	13624295	Glia maturation factor beta	0.48
Cyb5a	11560046	Isoform Short of Cytochrome b5	0.48
Anxa2	9845234	Isoform Short of Annexin A2	0.48
Pgm2	169642489	Phosphoglucomutase 2	0.48
Mdh1	15100179	Malate dehydrogenase, cytoplasmic	0.47
Akr1b3	6978491	Aldo-keto reductase family 1, member B3 (aldose reductase)	0.47
Cmpk1	71043752	Cytidine monophosphate (UMP-CMP) kinase 1	0.47
Eno1	158186651	Alpha-enolase	0.47
Calu	76559925	Calumenin isoform b	0.47
Pafah1b2	40254624	Platelet-activating factor acetylhydrolase 1b subunit 2	0.45
Ddah1	11560131	N(G), N(G)-dimethylarginine dimethylaminohydrolase 1	0.44
Anxa1	6978501	Annexin A1	0.42
Tagln2	61557028	Transgelin-2	0.42
Idi1	16758306	Isopentenyl-diphosphate delta-isomerase 1	0.41
Mgll	19923092	Monoglyceride lipase	0.41
Gsta4	157820217	Glutathione S-transferase alpha-4	0.41
Pygb	158187544	Brain glycogen phosphorylase	0.41
Actr1b	166157502	ARP1 actin-related protein 1B, centractin β	0.41
S100a11	51854249	S100 calcium binding protein A11	0.41
Cotl1	157823483	Coactosin-like protein	0.41
Xpo1	29789299	Exportin-1	0.38
Fdps	13929206	Farnesyl diphosphate synthase	0.38
Dstn	75991707	Destrin	0.37
Serpinb6a	40018548	Serine (or cysteine) peptidase inhibitor, clade B, 6a	0.35
Lasp1	14249130	LIM and SH3 protein 1	0.33
Gstp1	25453420	Glutathione S-transferase pi 1	0.33
Zyx	209915566	Zyxin	0.33
Akr1c14	19924087	Aldo-keto reductase family 1, member C14	0.31
Tagln	13928744	Transgelin	0.27
Cat	6978607	Catalase	0.20
	28849947	LOC286987 Hemiferrin	0.10
Tmsb4x	13592119	Thymosin beta-4	0.09

### SRM based proteomics

For further validation of our results with an independent method, we used selective reaction monitoring (SRM) mass spectrometry, which uses a triple-quadrupole mass analyzer for the sensitive detection of low-abundant proteins [23], [24]. To quantify the endogenous proteins, we added synthetic, heavy stable isotope-labeled peptides at a known concentration to each sample and calculated relative protein abundance by ratioing the intensity of the endogenous peptide to the respective heavy peptide. We used SRM to validate the levels of 27 proteins in the CE-enriched LDs and TAG-enriched LDs that were selected from the TMT based proteomic analysis and for potential interest. SRM analysis of the LD proteome confirmed the expression of 24 of the proteins associated with the LDs (**Table 3**). We also sought to compare the relative levels of expression on CE-enriched to TAG-enriched LDs. Previously mentioned Vdac1 and 2 were found to be elevated in CE-enriched LDs compared to TAG-enriched LDs, however not to the same extent. Using 2 peptides for Vdac1, levels measured by SRM were 1.82 and 1.74-fold higher in CE-enriched LDs compared to the 8-fold by TMT methods or using spectral counting (data not shown). Vdac2 was found to be 1.76-fold higher in CE-enriched LDs by SRM compared to 4.44-fold higher by TMT methods. Several proteins were enriched in CE LDs, such as sterol carrier protein (Scp2), which was elevated 4.38-fold in CE-enriched LDs by SRM compared to 1.19-fold by TMT methods, heat shock protein 1 (Hspd1), which was elevated 4.21-fold compared to 1.63-fold, Scarb1 elevated 2.34-fold compared to 6.28-fold, and Vim elevated 2.23-fold compared to 4.93-fold. Several proteins that were found to be elevated in CE-enriched LDs by SRM were found to be lower by TMT methods or using spectral counting (data not shown), such as annexin (Anxa1), which was elevated by 4.03-fold in CE-enriched LDs by SRM compared to 0.42-fold by TMT methods, and rab GDP-dissociation inhibitor alpha was elevated by 4-fold by SRM compared to 0.68-fold by TMT methods. In addition, lipid-binding protein perilipin (Plin1), which was not detected by TMT MS, was also not detected using SRM, whereas Rab8A, which was also not detected by TMT MS, was identified using SRM and found to be elevated in CE-enriched LDs.

**Table 3:** Comparison of select LD-associated proteins detected by SRM and TMT MS proteomics.

		SRM	TMT
Peptide	Gene symbol	CE/TAG	CE/TAG
IGGIFAFK	Scp2	4.377	1.189
DIGNIISDAMK	Hspd1	4.205	1.625
GLGTDEDTLIEILTTR	Anxa1	4.034	0.420
FVSISDLFVPK	Gdi2	4.000	0.683
VLEALLPLK	Fasn	2.456	1.035
YFPDMFPIK	Scarb1	2.336	6.277
ETNLESLPLVDTHSK	Vim	2.232	4.925
ASNGDAWVEAHGK	Hspa9	2.079	1.275
LTLSALLDGK	Vdac1	1.819	8.000
LTLSALVDGK	Vdac2	1.764	4.438
VTQSNFAVGYK	Vdac1	1.736	8.000
LLLIGDSGVGK	Rab8A	1.507	ND
TEFLSFMNTELAAFTK	S100a11	1.493	0.406
ILLAELEQLK	Vim	1.336	4.925
INVNEIFYDLVR	Rap1a	1.323	1.464
DISTNYYASQK	Hsp90b1	1.018	0.758
DLGYVPLVSWEEAK	Hsd3b1	0.918	6.063
LFFLDLK	Slc27a1	0.914	1.189
ELEEIVQPIISK	Hspa5	0.882	1.320
TFVSITPAEVGVLVGK	Pfn1	0.737	0.536
SLNILTAFR	Erp29	0.728	0.841
VLQATVVAVGSGGK	Hspe1	0.677	1.932
SELLVDQYLPLTQK	Plin2	0.659	1.932
VDYGGVTVDELGK	Pebp1	0.658	0.785
APVPTGEVYFADSFDR	Canx	0.656	3.249
LSVSWVEWK	Plin2	0.352	1.932
GLDHLEEK	Plin1	ND	ND
TLRPALVGVVK	Grpel1	ND	5.657
FFEDYGLFMR	Trap1	ND	0.25

ND, not detected.

## Discussion

The goal of our study was to compare differences in LD proteins between CE-enriched and TAG-enriched LDs. Previous studies have used various cell or tissue models to examine the LD proteome; however, these various cell types contain different cytosolic proteins, metabolic, and structural proteins, making it difficult to compare the results between studies. We have addressed this issue by performing our studies in one cell type, primary granulosa cells, and determined whether there is a preference toward certain LD-binding proteins based on LD composition. In using primary granulosa cells, our study is the first to examine LD proteins in a steroidogenic cell, which may provide a unique insight in LD proteins involved in steroidogenesis. Granulosa cells were incubated with either HDL or FA to induce the formation of CE-enriched LDs or TAG-enriched LDs, respectively. Peptide samples were labeled using TMT, an isobaric tag covalently linked to each lysine side chain and N-terminal group of a peptide. The labeled peptides were then mixed with unique tags bound to each biological sample, separated by LC, and analyzed using MS tandem mass spectrophotometry (MS/MS) [25]. There were 61 proteins that were elevated in CE-enriched LDs, whereas 40 proteins were elevated in TAG-enriched LDs.

There were 379 proteins detected by TMT based proteomics that were found to be associated with LDs. These proteins included proteins previously found on LDs, including membrane trafficking proteins annexin 2, Rab5c, and Rab8a, structural proteins vimentin, α-tubulin, and β-tubulin, chaperone proteins HSPa8, HSPd1, GRP78, HSP70, and HSP90, as well as LD protein ADRP (Plin2) and Vdac1 (for list see [1], [13], [14]). The presence of these proteins found in multiple screens suggests an importance in LD structure or function. In addition to these proteins, our screen detected several proteins that have previously not been identified in proteomics screens and were elevated in CE-enrich LDs, including Marcks-related protein (Marcksl1), vesicle-associated membrane protein-associated protein A (Vapa), and general vesicular transfer factor p115 (Uso1). Given that most proteomics screens involve TAG-enriched LDs, these proteins may represent unique LD-binding proteins that have a preference for CE-enriched LDs, or may be unique to LDs formed in granulosa cells; further analysis of these unique proteins is required to determine their function on the LD. A recent study using photoreactive sterol probes in combination with quantitative mass spectrometry to map cholesterol-protein interactions identified 250-cholesterol-binding proteins, which included receptors, channels and enzymes [26]. Our screen detected 34 similar proteins on CE-enriched LDs, including Vdac 1 and 2, 17-β hydroxysteroid dehydrogenase, Vapa, Scarb1, and solute carrier family 27 (Slc27). Proteins detected on the CE-enriched LDs but not found on the list of direct cholesterol-protein interactions might not directly bind to cholesterol moieties, perhaps instead interacting with proteins surrounding the LD or interacting with the phospholipid monolayer surrounding the LD.

Differences exist within the type of proteins found on LDs within different cells types, with one example being the perilipin family. Nascent LDs are coated with proteins during the budding process and while in the cytosol, primarily by perilipins. Members of the perilipin family have been found to be uniquely expressed in tissues [27]. Plin1 is an important regulator of lipid storage and is predominantly found on adipocytes and steroidogenic cells [28]–[30]. Plin2 and Plin3 are widely expressed in most cell types [31], [32]; Plin4 is found in white fat, skeletal muscle and the heart [33]–[35]; and Plin5 is found in the heart, brown fat, adipose tissue, liver, and skeletal muscle [32]. In our study, Plin2 appears to be the predominant Plin in granulosa cells (adipose differentiation-related protein, ADRP), which supports a previous finding of ADRP in primate granulosa cells [36]. A recent study demonstrated that different perilipin family members were found to preferentially associate with either TAG-enriched LDs or CE-enriched LDs [37].

Of the detected proteins, Vdac1 and 2 are particularly notable, due to recent findings of their involvement in lipid transfer within the mitochondria. VDACs are the most abundant mitochondrial outer membrane (MOM) proteins and serve as a large channel which functions in the pathway for mitochondrial respiratory substrates to cross the MOM, thus functioning as global regulators of mitochondrial function [38], [39]. Interestingly, Vdac1 was found to interact with steroidogenic acute regulatory (StAR) protein to facilitate the transfer of cholesterol into the inner mitochondrial membrane [40] for steroidogenesis. Therefore, our findings that Vdac appears to be present at higher levels on CE-enriched LDs compared to TAG-enriched LDs may signify a role of VDAC in binding of CE-enriched LDs or facilitation of CE movement into the mitochondria. In a similar fashion, vimentin, an intermediate filament that has been found to be associated with LDs [1], [3], [15] and involved in steroidogenesis [41], was increased in CE-enriched LDs. Ablation of vimentin in mice decreases movement of cholesterol to the mitochondria in adrenal and ovaries resulting in decreased corticosterone and progesterone production [42]. Several steroidogenic enzymes were detected in our proteomics analysis, including 3β-hydroxysteroid dehydrogenase/δ5-4 isomerase type 1 and 2 (Hsd3b1 and Hsd3b2), which were elevated in CE-enriched LDs compared to TAG-enriched LDs, suggesting that these enzymes might act directly upon the LD for steroidogenic activity. Further studies involving the role of steroidogenic enzymes at the LD are warranted.

A common challenge in analyzing LD proteomics is the presence ER, Golgi, and mitochondrial proteins. There can be uncertainty as to whether these proteins are actually LD-associated proteins or whether they are detected as a result of organellar contamination. Previous studies have shown that there are close associations between LDs and the mitochondria and ER, which may make separation of the LDs from the organelles difficult [13], [43]–[45]. In our studies, LD formation involved the incubation with cAMP, which is known to alter mitochondrial movement and to favor a close association with LDs. Although we did not observe mitochondria or membrane fractions contaminating our LD preparations when examined by EM, it is possible that mitochondrial proteins were released during disruption of cells for LD isolation and that this led to contamination of the LDs. With this caveat, the detection of mitochondrial proteins in the proteome of LDs suggests that they might be involved in facilitating LD-mitochondria interactions and steroidogenesis. Recent evidence suggests that several resident organellar proteins may provide a physical link between the organelle and the LD. Deletion of yeast Fld1, human orthologue of seipin, which resides in the ER, was found to lead to abnormal LD behavior, suggesting it plays a functional role as a LD scaffolding protein [46]. Numerous studies have identified the presence of Rab proteins, GTPases involved in membrane trafficking, to be present in LD proteomic screens [1], [3], [5], [14], [47]. Plin5 was found to provide a physical and metabolic linkage between LDs and mitochondria [43]. Whether or not these proteins are legitimate LD-associated proteins will require further deletion studies to determine the effects on LD intracellular localization and behavior.

To our knowledge, this is the first proteomics study to compare LD proteins from CE-enriched LDs with TAG-enriched LDs within the same cell type. These results were found by using a combination of TMT labeling and LC/MS based proteomics, which has been shown to be useful in determining a global protein population and in making comparisons between two samples. While the combination is powerful in identifying proteins, two previous studies indicate that the method failed to detect about 50% of the total proteins found within the cell [48], [49]. In addition to shotgun proteomics, SRM enables the quantitative comparison of protein concentration between samples [23], [24], [50], as opposed to shotgun spectral counting, which can only provide estimates of protein concentration differences between samples, or TMT, which generally provides relative differences in protein concentrations and is often complicated by the co-isolation of a second or third precursor ion that can distort the true quantification. This is the first time, to our knowledge, that SRM has been applied to LD proteins. This approach was used to validate the presence of select proteins on the LD and allows us to analyze the abundance of the protein in a much more quantifiable manner than a spectral counting approach, which is known to provide only an approximation of protein abundance [51]. The SRM approach was able to detect 92.6% (25/27) of the selected peptides found in the TMT MS approach. The SRM approach may be more sensitive, as Rab8A, a protein involved in endocytic and exocytic pathways and reported to bind LDs [1], was detected on the LD by SRM, but not detected by TMT MS proteomics. Future comparisons of LD proteins between two sets of samples should consider the use of SRM analysis.

In summary, our study has compared LD-associated proteins in CE-enriched and TAG-enriched LDs isolated from granulosa cells incubated with HDL or FA, respectively. To our knowledge, this is the first study to examine LD-associated proteins from CE-enriched LDs in a steroidogenic cell and to compare the differences in CE-enriched and TAG-enriched LDs. Using tandem mass tags based proteomics, we found 61 proteins elevated in CE-enriched LDs and 40 proteins elevated in TAG-enriched LDs with 278 proteins in similar amounts. SRM was used to further validate these results, which is the first time this technique has been applied to LD-associated proteins. These results help elucidate and create a better understanding of the proteins surrounding CE-enriched LDs in granulosa cells and highlight how differences in lipid composition can influence the proteins trafficking to LDs.

## Supporting Information

Table S1 **List of peptides and transitions used for selected reaction monitoring mass spectroscopy.**
(XLSX)
